# Improved Outcomes in Congenital Insensitivity to Pain with Anhidrosis (CIPA) via a Multidisciplinary Clinic Model

**DOI:** 10.3390/jcm14155258

**Published:** 2025-07-24

**Authors:** Moshe Shmueli, Galina Ling, Siham Elamour, Yaron Weisel, Shalom Ben-Shimol

**Affiliations:** 1 Faculty of Health Sciences, Ben-Gurion University of the Negev, Beer-Sheva 84101, Israel; galinali@clalit.org.il (G.L.); sihame@clalit.org.il (S.E.); yaronweisel@gmail.com (Y.W.); 2 The Pediatric Day-Care Unit, Soroka University Medical Center, Beer-Sheva 84101, Israel; 3 Department of Orthopedic Surgery, Soroka University Medical Center, Beer-Sheva 84101, Israel; 4 Pediatric Infectious Diseases Unit, Soroka University Medical Center, Beer-Sheva 84101, Israel

**Keywords:** multidisciplinary clinic, CIPA, bundle, judicious antibiotic use

## Abstract

**Background**: Congenital insensitivity to pain with anhidrosis (CIPA) is a rare genetic disorder, often leading to injuries and serious infections. In 2018, we established a multidisciplinary clinic (MDC) to provide structured, proactive care. We assessed the MDC’s impact on hospitalizations, surgeries, and infection rates. **Methods**: A retrospective study of genetically confirmed CIPA patients, treated from 2014 to 2024. Data from electronic medical records were compared between the pre-MDC (2014–2017) and post-MDC (2018–2024) periods. The core MDC team includes an infectious disease specialist, orthopedic surgeon, and nurses. The patients are stratified according to their carriage of resistant organisms and are managed using strict infection control measures. Follow-ups are scheduled routinely or as needed. Treatment is guided by clinical findings and culture results. **Results**: A total of 59 patients were included in the study. The baseline age did not differ significantly between the two periods. Hospitalization rates declined by 30.7% (from 57.7 to 40.0 per 1000 days), and clinic visits decreased by 42.9% (25.5 to 14.6). Overall surgical rates remained stable (2.8 to 2.7), with a 61.9% decrease in eye surgeries and a 130.5% increase in elective tooth extractions. Infection rates increased by 52% (from 6.6 to 10.1 per 1000 days). **Conclusions**: The implementation of the MDC bundle led to reduced hospitalizations, clinic visits, and eye surgeries, alongside the increased use of elective tooth extractions and culture testing. Closer monitoring and early infection management contributed to fewer severe complications. These findings support the value of structured, proactive multidisciplinary care in improving outcomes for children with CIPA.

## 1. Introduction

Congenital insensitivity to pain with anhidrosis (CIPA) is a rare autosomal recessive disorder affecting the autonomic and sensory nervous systems, with an estimated prevalence of approximately 1 in 25,000 individuals [1,2]. First described in 1951, it is notably more prevalent in regions with high rates of consanguineous marriages [2,3,4,5]. The disease results from loss-of-function mutations in the NTRK1 gene (1q21-q22), which encodes a receptor essential for nerve growth factor (NGF)-dependent sensory neuron survival [6,7,8]. These mutations cause the loss of myelinated Aδ and unmyelinated C fibers, impairing pain and temperature sensation, while preserving touch and pressure perception [6].

Clinically, CIPA presents in infancy with unexplained febrile episodes, due to impaired thermoregulation and anhidrosis. The absence of pain perception results in chronic, often self-inflicted injuries, such as burns and wounds, especially on the extremities, lips, and tongue, frequently following tooth eruption [9,10]. Motor and cognitive developmental delays are common [3,9].

Self-mutilation, a hallmark of CIPA, manifests as finger biting, oral ulcers, corneal injuries, painless fractures, orthopedic deformities, deep ulcers, and osteomyelitis (OM), predominantly affecting the limbs [1]. Chronic osteomyelitis in CIPA patients is often associated with pathological fractures, mainly in the limbs, but also in facial bones (e.g., mandible), posing significant treatment challenges. Children with CIPA commonly require hospitalization across multiple specialties, including orthopedics, oral and maxillofacial surgery, general pediatrics, and infectious diseases. Effective management demands a holistic approach to prevent and address complications.

The Soroka University Medical Center (SUMC), a tertiary referral center in southern Israel, offers a unique setting for studying CIPA, due to the provision of services to the local Bedouin population. This population is characterized by a high rate of consanguineous marriages, resulting in a correspondingly high prevalence of CIPA [5,11,12].

This context underscores the urgent need for effective treatment strategies and tailored management approaches for CIPA patients. As there is no definitive cure, treatment focuses on preventing injuries and managing bone and joint infections. Currently, no standardized guidelines exist for this rare disorder; thus, effective care depends largely on patient education and family training [13]. A possible solution to this challenge is fostering collaboration among health professionals within the hospital to create an integrated, centralized care framework. Multidisciplinary clinic (MDC) models have been successfully implemented in regard to chronic pediatric conditions, such as cystic fibrosis, spina bifida, and congenital heart disease, demonstrating efficacy in enhancing care coordination and improving patient outcomes [14,15].

Before the establishment of the multidisciplinary clinic (MDC) in 2018, patients with CIPA experienced frequent hospitalizations, primarily relying on pediatric wards for orthopedic and specialist care. Day hospital clinic visits were sporadic and driven by individual physician decisions, either in the community or during hospitalization. The absence of a coordinated care system led to prolonged lapses in follow-up, during which many patients re-presented with severe complications, such as chronic wounds, infections, major surgical needs, and, in some cases, fatal outcomes.

The unique challenges described above led to the establishment of a multidisciplinary clinic (MDC) at our hospital in January 2018. Adopting a structured, proactive management approach, the clinic integrates specialists from multiple disciplines to provide coordinated care. We implemented patient cohorting and routine screening for resistant pathogens, alongside scheduled follow-ups and continuous clinical monitoring to enable early infection detection and timely intervention. This multidisciplinary model aims to reduce hospitalizations, minimize complications, and improve patients’ quality of life.

Our objective was to evaluate the impact of a multidisciplinary, team-based approach on hospitalizations, surgical interventions, and infection rates in children with CIPA by comparing the outcomes before and after the establishment of the MDC. This study aims to contribute to the development of evidence-based strategies for optimizing care in regard to this rare disorder.

## 2. Methods

### 2.1. Study Design

This retrospective cohort study was conducted at the Soroka University Medical Center (SUMC). Data were extracted from electronic medical records to compare the patient outcomes before and after the establishment of the CIPA–MDC in January 2018. The study was approved by the SUMC Helsinki Committee on 18 October 2024 (Approval No. SOR-0256-24).

### 2.2. Study Population

The study population included all the patients diagnosed with CIPA and treated at the SUMC between 2014 and 2024. The inclusion criteria required a confirmed clinical and genetic diagnosis of CIPA. The pre-MDC cohort (2014–2017) included all the patients who were under active follow-up at the SUMC prior to the establishment of the multidisciplinary clinic. The post-MDC cohort (2018–2024) included two groups: (1) patients who began follow-up before the MDC was established and continued to be treated beyond age 18 or until the end of follow-up, and (2) patients who were born or diagnosed with CIPA after 2018 and began treatment during the period in which the MDC was in operation. This study design enabled longitudinal analysis of patients transitioning across care models, while also allowing the inclusion of new patients during the MDC’s period of operation. Patients who died or were lost to follow-up before 2018 were excluded from all the analyses.

Since the SUMC serves as the primary tertiary center in the Negev region of southern Israel, where all patients with CIPA receive treatment, with minimal visits to other medical centers in Israel, the comprehensive medical records from our hospital enabled incidence rate calculations to be conducted of hospitalization and surgical interventions.

Patients with incomplete medical records were excluded from the analyses.

### 2.3. Pediatric Multidisciplinary Clinic for Congenital Insensitivity to Pain

The MDC for CIPA follows a structured and proactive approach to patient care, focusing on several key components to optimize outcomes for affected children. The clinic employs cohorting strategies to ensure the delivery of efficient and coordinated care, grouping patients based on their specific needs and conditions (Table 1).

Active screening for the carriage of antibiotic-resistant pathogens is routinely conducted to identify and manage potential infection risks at an early stage. Patients who carry antibiotic-resistant bacteria are detected and treated at a dedicated facility, while children without such carriage are treated in a different area, by different staff. Strict infection control measures are enforced in the MDC, including adherence to hand hygiene protocols and the use of gowns and gloves when treating all patients who are known to carry antibiotic-resistant bacteria.

Patient follow-ups (in both cohorts) are scheduled both based on urgency and periodically, ensuring continuous monitoring, even when immediate concerns are not present. On average, patients visit the clinic once every two weeks.

The core multidisciplinary team consists of an infectious disease specialist and an orthopedic surgeon, who play key roles in infection prevention and clinical management. The orthopedic surgeon evaluates patients during clinic visits, facilitates surgical preparation, and schedules procedures as needed. The infectious disease specialist regularly reviews culture results to optimize antibiotic therapy, ensuring timely and appropriate treatment adjustments. Additional specialists, including ophthalmologists, oral and maxillofacial surgeons, plastic surgeons, psychiatrists, and other consultants, are involved on a case-by-case basis. A social worker is an integral team member, providing essential support to families who are coping with the challenges of living with CIPA.

At each visit, all the patients undergo a physical examination. Visible abscesses and wounds are routinely cultured, with treatment decisions, including antibiotic therapy, hospitalization, or surgical intervention, made based on clinical and microbiological findings. Additionally, laboratory monitoring, including blood counts and C-reactive protein (CRP) levels, is regularly performed to assess infection activity.

Other aspects of the MDC include monitoring the physiological and nutritional status of all the patients, including the need for vitamin treatment, iron supplementation, or other interventions.

### 2.4. Pre- and Post-MDC Visits and Hospitalizations

The number and duration of hospitalizations (hospitalizations episodes and hospitalization days, respectively) and MDC visits were meticulously recorded. The total number of hospitalization days and hospitalizations was then normalized to 1000 follow-up days. The follow-up period concluded upon the patient’s death or discontinuation of follow-up at the dedicated clinic. Only hospitalizations in the general pediatric wards, the pediatric surgical ward, or the pediatric orthopedic ward were included in the hospitalization count.

Since the multidisciplinary clinic was established on 1 January 2018, all hospitalizations and interventions performed before this date (2014 through 2017) were considered to have occurred during the pre-MDC period, while those occurring afterward (2018 through to 2024) were classified as the post-MDC period. To assess the impact of follow-up at the dedicated multidisciplinary clinic on the quality of care, treatment adherence, and patient compliance, the number of clinic visits was also documented and analyzed.

### 2.5. Surgical Interventions

To assess the number of surgical interventions, patients’ medical records, including all surgical reports, were retrieved and analyzed by a specialist. The number of surgeries performed before and after the establishment of the multidisciplinary clinic was documented. A specialist classified the surgeries into five main categories: orthopedic surgeries, ophthalmologic procedures, oral and maxillofacial surgeries, plastic surgeries, and other.

### 2.6. Infections

For the purposes of this study, we included only osteomyelitis, septic arthritis, other deep tissue infections, and bacteremia as infections. Infection episodes were defined both clinically by the treating infectious disease specialist and by a positive culture from a bone, synovial fluid, skin and deep tissue abscess, pus from a skin wound, or a blood culture. We included only one episode per patient per week for the same pathogen or the same infection site. We excluded cases involving screening cultures (e.g., nasal cultures for *Staphylococcus aureus* or rectal cultures for ESBL-producing bacteria), as well as urine cultures.

### 2.7. Data Collection

Data were retrospectively extracted from the hospital electronic medical records, encompassing a range of demographic, clinical, and outcome-related variables. The demographic data included age, gender, and ethnicity. Quantitative measures included the planned number of hospitalizations, the number of visits to the MDC, and the number of interventions, both before and after the establishment of the clinic.

### 2.8. Statistical Analysis

Descriptive statistics were used to summarize the demographic and clinical characteristics of the study population. Categorical variables were presented as frequencies and percentages, while continuous variables were reported as the mean ± standard deviation (SD) when normally distributed. Univariate analyses were performed using SPSS version 29 to compare the hospitalization rates, clinic visits, and surgical procedures across different time periods, before and after the establishment of the multidisciplinary clinic (MDC) for CIPA patients. Incidence rates, rate ratios (RRs), and their corresponding 95% confidence intervals (CIs) were calculated to assess differences between the groups. Subgroup analyses were also conducted to compare different types of surgeries, based on predefined categories. Statistical significance was defined as *p* < 0.05.

## 3. Results

During the study period, 59 patients met the inclusion criteria and were analyzed. Of these, 35 were included in the pre-MDC cohort (2014–2017). The post-MDC cohort (2018–2024) included 58 patients: 34 who transitioned from the pre-MDC period and remained in follow-up (1 patient died), and 24 newly diagnosed patients, who entered treatment during the MDC period. All the patients (100%) were of Bedouin ethnicity, with 33 (56%) males. The mean age at the start of follow-up was 8.15 ± 8.98 years for the pre-MDC period and 4.72 ± 10.16 years for the post-MDC period (n = 51), a difference that was not statistically significant (*p* = 0.130).

### 3.1. Hospitalization Episodes and Days

The pre-MDC period ranged between 0 and 1440 days (median 660 days, mean of 657 ± 609 days). During this period, the range of hospitalization episodes (days) was between 0 and 275 days (mean of 37.9 ± 63.9 days) (Table 2).

The post-MDC period ranged between 0 and 2520 days (median 1800 days, mean of 1616 ± 861 days). During this period, the range of hospitalization episodes (days) was between 0 and 527 episodes (mean of 63.3 ± 96.9 days).

Overall, comparing the pre- and the post-MDC periods, the hospitalization episodes rate per 1000 days declined by 30.7%, from 57.7 to 40.0, *p* < 0.001 (Table 3).

Notably, one patient was reported to have a very poor level of compliance in regard to treatment, with a lack of adherence to scheduled MDC visits. This patient had 36 days of hospitalization during the pre-MDC period, compared with 527 days of hospitalization during the post-MDC period. Thus, the calculated reduction in the hospitalization episodes rate per 1000 days for all of the other 58 patients was 39.7%, from 58.6 to 35.4, *p* < 0.001.

### 3.2. Day Hospitalization Clinic and MDC Visits

During the pre-MDC period, the range of visits (per patient) to the day hospitalization clinic was between 0 and 96 days (mean of 16.8 ± 25.0 days). During the post-MDC period, the range of visits (per patient) was between 0 and 116 days (mean of 23.5 ± 24.6 days).

Overall, comparing the pre- and the post-MDC periods, the clinic visits rate per 1000 days declined by 42.9%, from 25.5 to 14.6, *p* < 0.001.

### 3.3. Surgical Interventions

During the pre-MDC period, the range of surgical interventions (per patient) was between 0 and 13 interventions (mean of 1.8 ± 3.5). During the post-MDC period, the range of surgical interventions (per patient) was between 0 and 25 interventions (mean of 4.4 ± 5.4 days).

Most surgical interventions were orthopedic surgeries, during both the pre- and the post-MDC periods. During the pre-MDC period, 80 of 107 (74.8%) surgery interventions were orthopedic surgeries, including 75 incision and drainage procedures (93.8% of all orthopedic surgeries) and 5 amputations. During the post-MDC period, 180 of 257 (70.0%) surgical interventions were orthopedic surgeries, including 160 incision and drainage procedures (88.9% of all orthopedic surgeries) and 20 amputations.

Other common surgical interventions included eye surgeries for corneal ulcer repair, with 16 surgeries during the pre-MDC period and 15 surgeries during the post-MDC period. Elective tooth extraction, performed by mouth and jaw surgeons, was documented 8 times during the pre-MDC period and 51 times during the post-MDC period. Plastic surgeries, including the amputation of fingers and the debridement of damaged tissues, were documented in regard to two episodes during the pre-MDC period and nine episodes during the post-MDC period. One patient underwent percutaneous endoscopic gastrostomy surgery, and one patient had a scalp hematoma drainage performed by general pediatric surgeons, both during the post-MDC period.

Overall, comparing the pre- and the post-MDC periods, the surgical intervention rate per 1000 days declined by 2.3%, from 2.8 to 2.7, *p* = 0.84. The respective reduction in the surgical intervention rate was 8.5% (*p* = 0.51) for orthopedic surgeries and 61.9% (*p* = 0.007) for eye surgeries. In contrast, the respective tooth extraction rate (per 1000 days) increased by 130.6%, from 0.23 to 0.53, *p* = 0.02.

The rates of plastic surgeries and other surgeries were low and did not exhibit a significant trend.

### 3.4. Infections

During the pre-MDC period, the number of infection episodes per patient ranged from 0 to 40 (mean 4.4 ± 8.1). During the post-MDC period, the range was between 0 to 123 episodes per patient (mean 16.4 ± 29.3).

Overall, the infection episode rate increased by 52%, from 6.6 to 10.1 per 1000 days (*p* < 0.001). Similarly, infections caused by Enterobacteriaceae (including *Escherichia coli*, *Klebsiella* spp., *Enterobacter* spp., and *Proteus* spp.) rose by 91%, from 1.0 to 1.9 episodes per 1000 days (*p* < 0.001).

## 4. Discussion

### 4.1. Clinical Interpretation

In this study, we report on our experience of operating a holistic multidisciplinary clinic (MDC) for children with CIPA and its impact on clinical outcomes. Following the establishment of the MDC, the hospitalization rates declined by 31%, accompanied by a 43% reduction in clinic visits, suggesting that the reduced hospital utilization was not offset by an increased outpatient volume.

The overall and orthopedic surgical intervention rates remained stable, while eye surgery rates (primarily corneal ulcer repairs) decreased significantly, by 62%. In contrast, elective tooth extractions increased by 131%. Infection rates rose by 52%, as expected in the context of more proactive detection and closer follow-up.

This differential pattern suggests that the MDC facilitated preventive, elective interventions, while reducing the need for urgent, complication-driven procedures, supporting the effectiveness of a structured, proactive care model in managing CIPA.

### 4.2. Comparison to Other MDC Models and Common Challenges

Multidisciplinary centers (MDCs) have been proven to be highly effective in managing complex and chronic pediatric conditions by integrating various medical specialties within a coordinated care framework. A prominent example is cystic fibrosis (CF), where dedicated CF centers have significantly improved outcomes through continuous monitoring, early infection detection, and the provision of comprehensive nutritional support [16,17,18]. Similarly, MDCs for pediatric oncology have enhanced patient survival by coordinating chemotherapy, surgery, and psychosocial care within a unified setting [19,20]. The success of such centers is often linked to structured follow-up protocols, proactive infection control, and seamless interdisciplinary collaboration. However, their effectiveness is limited in regard to healthcare systems with poor infrastructure, inadequate funding, staffing shortages, or fragmented care, where continuity and coordination are difficult to maintain [21]. In southern Israel, where CIPA is disproportionately prevalent among low socioeconomic status populations, these challenges are particularly acute and underscore the need for tailored, well-supported care models.

### 4.3. Implementation of the MDC for CIPA Patients

The establishment of the MDC for CIPA patients led to a significant reduction in hospitalization rates, an indicator of both clinical severity and systemic challenges. In this population, prolonged admissions often reflect advanced disease due to delayed care, antibiotic-resistant infections, or severe injuries [3,4,13]. These issues frequently stem from patients being lost to follow-up, neglect, or fragmented care, as the need for specialized consultations (e.g., with orthopedic surgeons) or to perform complex interventions is prompted by clinical deterioration. Improved adherence to MDC visits enabled earlier detection of complications and more timely interventions, reducing the need for urgent hospitalizations and complex procedures. Importantly, this structured approach also led to a decrease in the overall number of clinic visits, by 43%, underscoring its efficiency and preventive value. This shift was seen also in surgical patterns, with fewer ocular procedures and more oral surgeries, mainly preventive tooth extractions. In CIPA patients, dental trauma and poor hygiene often lead to severe complications. Early extractions helped prevent complex interventions, reflecting a move from reactive to preventive care.

A key MDC feature was systematic infection screening with routine cultures from relevant sites (e.g., bone, joint fluid, abscesses, blood). Paradoxically, despite detecting more infections, this enabled timely, targeted treatment that ultimately reduced the need for interventions and hospitalizations. Combined with frequent follow-up and patient/family education, infection control was likely the most impactful factor in improving outcomes.

### 4.4. Study Limitations

This study has several limitations. As a retrospective, single-center analysis, its generalizability to other regions or healthcare systems is limited. The absence of a control group and reliance on medical records introduce potential documentation bias. Additionally, secular trends in care over time may have influenced outcomes independently of the multidisciplinary approach. While the cohort is relatively large for this rare condition, the findings may not apply to broader populations. We accounted for age, an important factor, in regard to complications and hospitalizations, but acknowledge that residual confounding may remain.

## 5. Conclusions

In conclusion, our findings underscore the impact of a proactive, multidisciplinary approach to CIPA management. The establishment of the MDC led to a significant reduction in hospitalizations and eye surgeries, while increasing the identification of active infections, highlighting the benefits of improved preventive care. The overall and orthopedic surgery rates remained stable, while the sharp increase in tooth extractions reflects a strategic shift toward elective, preventive interventions. We suggest that an active, multidisciplinary care model not only helps prevent complications, but also facilitates timely, less invasive treatments, ultimately enhancing patient outcomes and quality of life. Future studies should explore long-term outcomes and replicability in other settings.

## Figures and Tables

**Table 1:** Multidisciplinary clinic (MDC) bundle: key interventions.

Type of Intervention	Details
Proactive approach	Every two weeks—MDC
	Scheduled appointments
Cohorting	Carriage status: sampling for antibiotic-resistant bacteria (AB-R ^1^; MRSA ^2^, ESBL ^3^, VRE ^4^)
	Separate areas for “clean” and “dirty/AB-R carriage”
	Separate staff for “clean” and “dirty/AB-R carriage”
	Avoidance of calling immunocompromised patients in regard to “CIPA day”
Infection control	Use of gown in “dirty” area
	Use of gloves in “dirty” area
	Strict adherence to hand hygiene guidelines
Staff	Orthopedic surgeon—routine
	Infectious disease specialists—routine
	Nurse (dedicated)—routine
	Additional staff—1 or 2 pediatrics residents
	Per need: ophthalmologist, oral and maxillofacial surgeon, plastic surgeon, psychiatrist; social work
Physical examination	Vital signs
	Mandatory full examination, including undressing, removal of all bandages, observation of wounds/lacerations/abscesses
Cultures	Review of previous results
	Active wounds/lacerations/abscesses, including superficial and deep tissue
	Blood cultures if febrile/toxic/hypotension/hypothermia
Imaging	As indicated
Laboratory	Review of previous results
	CRP for active cases/surveillance
	Other lab exams as needed, such as blood count, electrolytes, ferritin, vitamin D levels, etc.
Decisions	Antibiotic treatment
	Hospitalization
	Next MDC–CIPA appointment
	Need for surgical interventions
	Other therapeutics (zinc level, vitamin A, vitamin D, iron, etc.)

^1^ AB-R: antibiotic resistant; ^2^ MRSA: *methicillin-resistant staphylococcus aureus*; ^3^ ESBL: *extended-spectrum beta*-lactamase-producing bacteria; ^4^ VRE: *vancomycin-resistant enterococcus*.

**Table 2:** Hospitalization, MDC clinic visits, and surgical intervention episodes.

	Pre-MDC (n/Days); 2014–2017; n = 38,790 Days	Post-MDC (n/Days); 2018–2024; n = 95,340 Days
Hospitalization range (days)	0–275	0–527
Hospitalization mean ± SD (days)	37.9 ± 63.9	63.3 ± 96.9
Clinic visits range (days)	0–96	0–116
Clinic visits mean ± SD (days)	16.8 ± 25.0	23.5 ± 24.6
Surgical interventions range (days)	0–13	0–25
Surgical interventions mean ± SD (days)	1.8 ± 3.5	4.4 ± 5.4
Infection episodes range (days)	0–40	0–123
Infection episodes mean ± SD (days)	4.4 ± 8.1	16.4 ± 29.3
Surgical interventions (overall)	107	257
Orthopedic surgeries—incision and drainage	75	160
Orthopedic surgeries—amputation	5	20
Eyes—corneal ulcer repair	16	15
Mouth and jaw—tooth extraction	8	51
Mouth and jaw—internal fixation of pathologic fracture	1	0
Plastic surgery—amputation of fingers, debridement	2	9
Other surgery (general, gastro)	0	2
Infections—overall	257	963
*Staphylococcus aureus*	90	244
Enterobacteriaceae *	39	183
*Enterococcus* spp.	17	49
*Pseudomonas* spp.	43	129

* *Escherichia coli*, *Klebsiella* spp., *Enterobacter* spp., and *Proteus* spp.

**Table 3:** Rates of hospitalization, MDC visits, surgical intervention and infection episodes per 1000 follow-up days among CIPA patients, comparing the pre-MDC and the post-MDC periods.

	Episodes per 1000 Days (Pre-MDC)	Episodes per 1000 Days (Post-MDC)	Rate Ratio (RR) (95% CI)	*p* Value
Hospitalization—total	57.7	40.0	0.693 (0.6586–0.7291)	<0.001
Clinic visits—total	25.5	14.6	0.5708 (0.5266–0.6188)	<0.001
Surgery—total	2.8	2.7	0.9772 (0.7802–1.2240)	0.84
Surgery—orthopedics	2.1	1.9	0.9154 (0.7037–1.1901)	0.51
Surgery—eyes	0.4	0.16	0.3814 (0.1886–0.7714)	0.007
Surgery—mouth and Jaw	0.23	0.53	2.3055 (1.1352–4.6826)	0.02
Surgery—plastic surgery	0.05	0.09	1.83 (0.3956–8.4735)	0.77
Infections—overall	6.6	10.1	1.52 (1.33–1.75)	<0.001
*Staphylococcus aureus*	2.3	2.6	1.10 (0.87–1.40)	0.80
Enterobacteriaceae *	1.0	1.9	1.91 (1.35–2.70)	<0.001
*Enterococcus* spp.	0.4	0.5	1.17 (0.68–2.04)	0.57
*Pseudomonas* spp.	1.1	1.4	1.22 (0.86–1.72)	0.26

* *Escherichia coli*, *Klebsiella* spp., *Enterobacter* spp., and *Proteus* spp.

## Data Availability

The data presented in this study are available on request from the corresponding authors.

## Abbreviations

CIPA	Congenital insensitivity to pain with anhidrosis
HSAN	Hereditary sensory and autonomic neuropathy
NGF	Nerve growth factor
OM	Osteomyelitis
SUMC	Soroka University Medical Center
MDC	Multidisciplinary clinic
CRP	C-reactive protein
RR	Rate ratio
CI	Confidence interval
SD	Standard deviation
SPSS	Statistical Package for the Social Sciences
